# Blastulation time measured with time-lapse system can predict in vitro viability of bovine blastocysts

**DOI:** 10.1371/journal.pone.0289751

**Published:** 2023-08-10

**Authors:** Carmen Huayhua, Misael Rodríguez, Jhorjhi Vega, Mario Briones, Lleretny Rodriguez-Alvarez, Edwin Mellisho

**Affiliations:** 1 Centro de Investigación en Tecnología de Embriones (CIETE), Animal Improvement Program, Universidad Nacional Agraria La Molina, Lima, Perú; 2 Department of Animal Science, Faculty of Veterinary Sciences, Universidad de Concepción, Chillán, Concepción, Chile; University of Connecticut, UNITED STATES

## Abstract

The objective of this study was to evaluate the time of blastulation monitored by time-lapse technology to predict in vitro viability of bovine blastocysts. This technology can be a powerful tool for bovine embryos selection with higher implantation capacity and competence. Also, in humans an early blastulation is associated with higher quality and pregnancy rate. Cumulus oocyte complexes (COCs) were matured for 20 to 22 h and then fertilized by co-incubation of COCs and spermatozoa (10,000 sperm per oocyte) for 18 h. Presumptive zygotes were placed individually in microwells, in droplets of commercial culture medium. The Primo Vision TL system (EVO+; Vitrolife) captured digital images of developing embryos every 15 minutes. The time frame from IVF to the start of blastulation (tSB) and to blastocyst development (tB) was recorded. After day 7.5, the blastocysts were in vitro culture for 48 h until day 9.5 after IVF to evaluate post hatching development. In vitro viability was evaluated at day 9.5: those with a diameter greater than 200 μm and a total cell count greater than 180 were classified as viable (value 1), while the rest were classified as non in vitro viable (value 0). The area under the ROC curve (AUC) was estimated to determine the predictive power of *in vitro* viability through blastulation time. In addition, binary logistic regression analysis was used to generate a mathematical model with morphokinetic variables that allow the best prediction of *in vitro* viability. In 13 sessions, the blastocyst production rate was 46.2% (96/208). The cut-off time to discriminate early or late blastulation was 149.8 h. The post-hatching development of the embryos with early blastulation was 63.3% (31/49), being statistically superior (p = 0.001) than the late blastulation group 14.9% (7/47). Likewise, the time of blastulation showed an accuracy of 90.8% (p < 0.001) in predicting *in vitro* viability of bovine blastocysts. In conclusion, the selection of blastocysts based on blastulation time (< 155 h) and blastocyst diameter measured on day 7.5 after IVF (> 180 μm) maximizes the *in vitro* viability.

## Introduction

For the world embryo industry, *in vitro* fertilization (IVF) is the most important innovation that allow the trade of genetics of various species [1]. Over 1.5 million *in vitro*-produced (IVP) bovine embryos were recorded in 2021 [2]. Although, low pregnancy rate (33.5%; [3]) and high proportion of chromosomal abnormalities (20 to 25%; [4]), increased pregnancy loss [5], abnormal placental development [6], heavier fetuses [7], higher rates of dystocia, congenital anomalies [8], stillbirths and neonatal mortality [9] have been suggested as a major cause of failure of IVP system in cattle. Therefore, non-invasive criteria are required to achieve an objective and more precise embryo selection [10].

Early embryonic development involves a series of orchestrated events between the first cell cleavages and the differentiation of the first cell lineages [11,12], leading to the differentiation of the inner cell mass into hypoblast and epiblast (blastocyst formation) important events for the implantation of the embryo in the uterus [13]. Also, in humans, blastulation timing is associated with mitochondrial content, chromosome status, and embryonic quality and competence [14–16], synchrony between embryo development and endometrial receptivity [17] and high precision of pregnancy rate (80%; [18]). In cattle, blastulation timing may have a high potential for embryo selection over other embryo morphologic parameters and may be a criterion for the selection of blastocysts to be transferred that can predict the embryo viability or competence.

The competence of the oocyte and/or embryo is of great importance for the in vitro embryo production programs, impacting on the successful establishment of pregnancy after transfer to recipients [19]. The traditional selection of in vitro produced bovine embryos is mainly based on morphological characteristics [20], though this classification is considered subjective and inadequate [21]. Time-lapse technology is a non-invasive method that allows obtaining continuous digital images to monitor embryonic development *in vitro* [22]. Studies in humans indicate that the selection of competent embryos using continuous monitoring technologies is the more accurate method to identify embryos with greater implantation capacity [22–24]. However, it is little used in bovine embryo transfer [25,26]. The objective of this work is to evaluate the value of blastulation timing monitored by time-lapse technology to select embryos with greater post-hatching development.

## Materials and methods

The Graduate School of the La Molina National Agrarian University did not request the approval of the ethics committee, since the research only used commercial frozen semen and ovaries collected at a local slaughterhouse. The chemicals were purchased from Sigma-Aldrich (St. Louis, MO, USA), and the *in vitro* embryo culture media from Vitrogen (YVF Biotech LTDA EPP, Sao Paulo, Brazil).

### Experimental design

**Fig 1:**
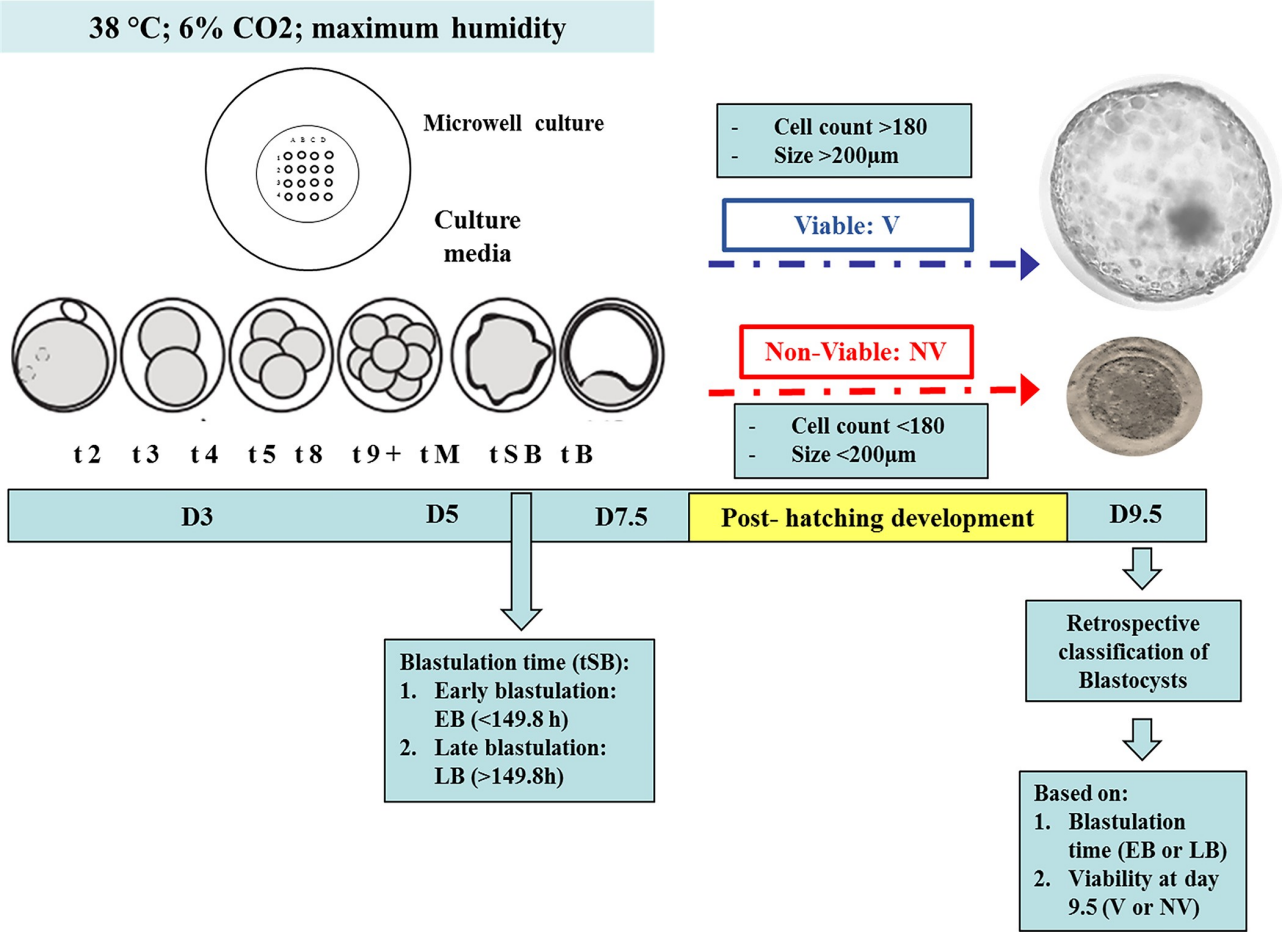
Schematic illustration of experimental design. Presumptive zygotes were cultured individually and embryonic development monitored with images captured by the Primo Vision TL system for a period of 7.5 days. At day 9.5 post IVF, blastocysts were classified according to their post-hatching development (V: Viable, NV: Non-viable).

Presumptive zygotes were cultured individually in microwell plates (16-microwell, Vitrolife, Gothenburg, Sweden). Embryo development was monitored with images captured by the Primo Vision TL system (EVO+; Vitrolife, Gothenburg, Sweden) for 7 days. The morphokinetic parameters were annotated: t2 (h) division time to 2 cells; t3 (h) division time to 3 cells; t4 (h) division time to 4 cells, t5 (h) division time to 5 cells, t8 (h) division time to 8 cells; t9+ (h) division time to 16 cells, tM (h) time to morula stage; tSB (h) time of starting blastulation, tB (h) time to blastocyst stage and tBX (h) time to expanded blastocyst stage. At day 7.5, morphological characteristics of blastocysts, embryo quality, developmental stage and blastocyst diameter were determined, using criteria described in the IETS manual [27]. In addition, on day 7.5, embryos were kept in individual culture, to assess their post-hatching development (in vitro viability) based on growth performance and diameter (Fig 1).

### *In vitro* embryo production

**Fig 2:**
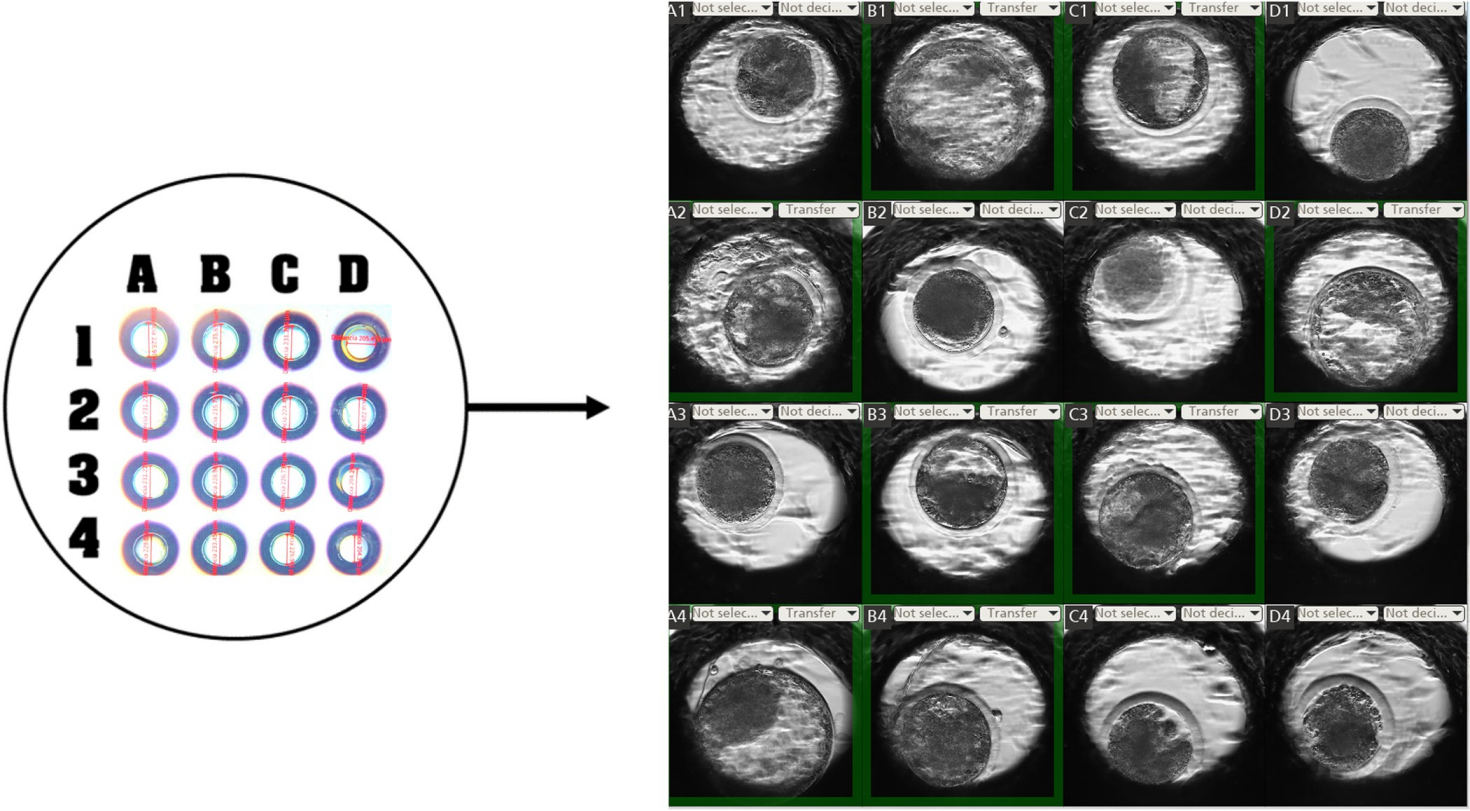
Individual culture to monitor embryonic development (Primovision®, Vitrolife, Sweden).

Ovaries were obtained from local abattoirs following the standard procedure described by Rodríguez et al. [28]. COCs in groups (10 to 12) were *in vitro* matured (IVM) in a drop (70 μL) of IVM medium (Vitrogen®, Brazil) for 20 to 22 h. Immediately after sperm selection, 10,000 motile spermatozoa per oocyte were used in IVF and co-incubated with COCs at 38°C in an atmosphere of 6% CO_2_ in air, similar to other authors [29,30]. After 18 h of IVF, presumptive zygotes were mechanically denuded by pipetting and then *in vitro* cultured (IVC) in IVC medium (Vitrogen, Brazil) at 38°C in 6% CO_2_ in air. During *in vitro* culture, 16 presumptive zygotes were individually (Fig 2) monitored using the Primo Vision TL® equipment (Vitrolife, Sweden) that takes images every 15 minutes from day 1 to 7.5 post IVF. On day 3 and 5 post IVF, culture media was refresh by changing 50% of the culture medium. The morphokinetics parameters (t2, t3, t4, t5, t8, t9+ tM, tSB, tB, tBX) were recorded during the culture of the embryos until day 7.5 post IVF. The starting point of IVF was considered as time zero (t0). Variables related to the duration of cell cycles were also determined and designated: duration of second cell cycle t3–t2, duration of third cell cycle t4–t3, duration of cell cycle between t5–t4, duration of cell cycle between t8–t5, duration of cell cycle between t9+–t8, duration of cell cycle between tM–t9+, duration of cell cycle between tSB–tM, duration of cell cycle between tB–tSB, duration of cell cycle between tBX–tB, which combines the concepts of cell cycle and synchrony (S1 Fig).

At the end of individual embryonic development monitoring (day 7.5 post IVF), each video was processed to establish embryonic division times. In addition, the images with calibrated measurements were saved in JPG format to be processed with the ImageJ software to measure the diameters of the embryos at tSB, tB and tBX times. At day 7.5, morphological characteristics of the blastocysts were determined; embryo quality, developmental stage and blastocyst diameter. In vitro viability was evaluated at day 9.5: those with a diameter greater than 200 μm and a total cell count greater than 180 were classified as viable (value 1), while the rest were classified as non in vitro viable (value 0).

As laboratory control, embryos were cultured in groups of 10 to 12 zygotes in 70 μL microdrops under mineral oil. On day 3 and 5 post IVF, a 50% change of the culture medium was performed and on day 3 and 7.5 post IVF the cleavage and blastocysts rate were evaluated, respectively.

For cell count, embryos were individually fixed in 1% paraformaldehyde in saline phosphate buffer for 30 minutes at 4°C. Subsequently, the fixed embryos were incubated individually at 37°C for 15 to 20 min in a dark environment for blastomere nuclei staining in drops of 10 μL of manipulation medium supplemented with 2 μL of DAPI solution (NucBlue™ Fixed Cell ReadyProbes™ Reagent, Thermo Fisher Scientific) and covered with mineral oil. Finally, embryos were mounted on a slide for counting the nuclei using a fluorescence microscope (Axioscope, Carl Zeiss, USA) with a magnification of 100x at a wavelength of 365 nm.

### Statistical analysis

Morphokinetics variables (t2, t3, t4, t5, t8, t9+ tM, tSB, tB, tBX) and cell cycle and synchrony (t3-t2, t4-t3, t5-t4, t8-t5, t9+-t8, tM-t9+, tSB-ttM, tB-tSB, tBX-tB) measurements were classified according to embryo viability and blastulation time and analyzed for normality of distribution using Kolmogorov–Smirnov test and for homogeneity of variance using Levene’s Test after which these variables were analyzed with ANOVA.

For exploring the relationship between two quantitative variables and one categorical variable we performed a scatterplot analysis with groups. Principal component analysis (PCA) was used for the multidimensional data set to emphasize variation and highlight strong patterns of morphokinetics and cell cycle variables and synchrony of embryonic development according to their in vitro viability (V = viable and N = non-viable) applying the same approach adopted by Mellisho et al. [26].

The predictive model of embryo *in vitro* viability proposed here was based on binary logistic regression to describe the dichotomous dependent variable of the blastocyst (viable = 1 and non-viable = 0). A set of independent morphokinetic variables were analysed with multiple regression. The logistic regression generated the coefficients, the standard errors and level of significance of the model for calculating the probability to predict the viability of the embryos, where values between 0.5 and 1 indicated blastocyst viability. To verify the predictive power of the algorithm, the following indicators were used: ROC-AUC (receiver operating characteristic analysis with determination of the area under the curve), percentage of correct predictions and omnibus tests. Statistical significance was determined at the P < 0.05 level. A rough guide for classifying the accuracy of a predictive model was 0.90–1 = excellent, 0.80–0.90 = good, 0.70–0.80 = fair, 0.60–0.70 = poor, 0.50–0.60 = fail. Statistical analysis was performed with the IBM SPSS Statistics program, version 20 (IBM, Armonk, NY, USA).

## Results

In this work, 923 viable COCs (quality 1 and 2) were used in 13 *in vitro* embryo production sessions (Table 1). The blastocyst rate obtained on day 7.5 post IVF and cultured in a time-lapse system (42.8%) was superior (p = 0.0001) to the laboratory control group (30.7%).

**Table 1:** Production of bovine embryos *in vitro* in a time-lapse system and culture in drops (laboratory control).

Treatment	Rep.	COCs (quality 1 and 2)	Cleavage rate (D3 post IVF) (x± SD)	Blastocyst rate (D7.5 post IVF) (x± SD)
Laboratory Control (in drops)	13	715	82.1± 5.2 ^a^	30.7± 3.1 ^a^
Microwell (Individually) Time-lapse system	13	208	83.7± 7.0 ^a^	42.8± 9.2 ^b^

a,b Different letters in the same column show significant differences (P<0.05). X: Mean, SD: Standard Deviation.

### Impact of blastulation time on embryonic development in vitro

Out of 208 embryos cultured in microwell culture system (time-lapse system), 174 (83.65%) had a first cleavage and 96 (42.8%) developed to the blastocyst stage. The 96 embryos that formed blastocysts reached mean values of t2, t3, t4, t5, t8, t9+ tM, tSB, tB and tBX at 29.41, 35.30, 43.21, 50.58, 65.74, 80.76, 109.66, 149.79, 164.42 and 171.98 h post IVF, respectively (see S1 Table). Also, they were retrospectively classified as viable (>200 μm and >180 cell count) 38/96 (39.58%) and as non-viable (<200 μm and <180 cell count) 58/96 (60.42%) (Table 2).

**Table 2:** Characteristics of *in vitro* cultured blastocyst according to *in vitro* viability determined by extended culture up to 9.5 days post IVF.

Viability *in vitro*	n	Blastulation time (tSB)		Blastocyst stage (tB)		Blastocyst (7.5 post IVF)
		Time (h)	Diameter (μm)	Time (h)	Diameter (μm)	Diameter (μm)
**Viable**	38	142.55±7.79 ^a^	155.36± 4.45 ^a^	159.75±8.76 ^a^	168.93±7.77 ^a^	209.29±17.19 ^a^
**Non-viable**	58	154.54±12.92 ^b^	154.81±4.60 ^a^	167.91±9.95 ^b^	167.21±7.53 ^a^	176.35±15.02 ^b^

a,b Different letters in the same column show significant differences (P<0.05).

In this study, of 96 embryos (96/208) that reached blastulation, 49/96 (51.04%) embryos showed early blastulation (<149.79 h) and 47/96 (48.95%) embryos showed late blastulation (>149.79 h). In Table 3, we observed that blastulation time is critical for *in vitro* viability, presenting high and significant viability the embryos with early versus late blastulation (63.27 vs 14.89%, respectively). The average blastulation timing for the 96 embryos resulted in 149.79 h.

**Table 3:** Characteristics of blastocyst cultured *in vitro* according to blastulation time.

Group	N	Blastulation time (h) (x± SD)	Blastocyst rate (7.5 post IVF)		Post hatching development (9.5 post IVF)		
			%	Diameter (μm)	%	Total count cell	Diameter (μm)
**Early**	49	140.01±6.48^a^	49 (100%)	204.04±19.59^a^	31 (63.27%) ^a^	268.03±72.97	335.60±73.51
**Late**	47	159.99±8.70^b^	47 (100%)	174.11±13.82^b^	7 (14.89%) ^b^	260.7±65.94	309.65±85.46

a,b Different letters in the same column show significant differences (P<0.05). X: Mean, SD: Standard Deviation.

**Fig 3:**
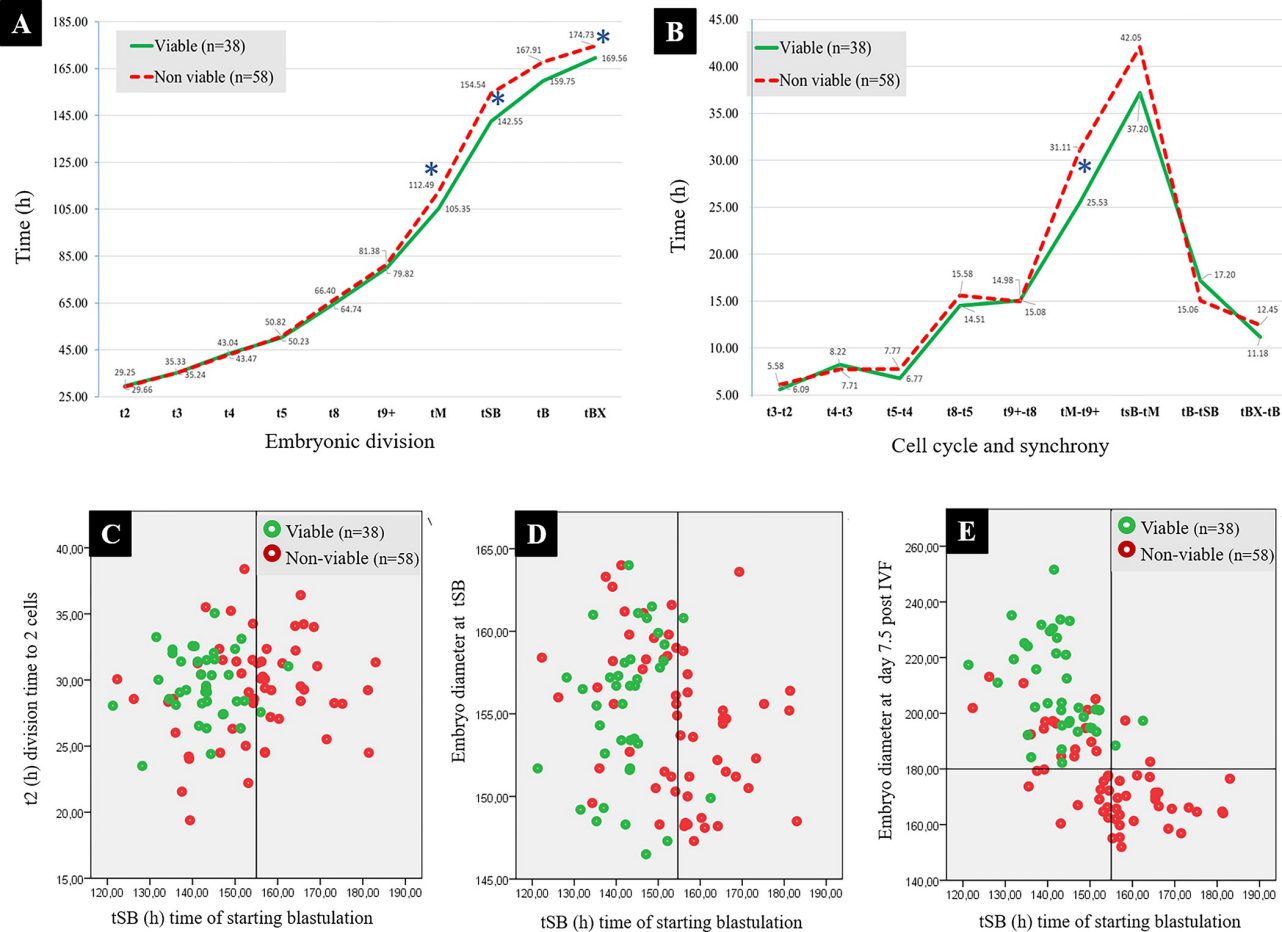
Bovine embryonic development in microwell culture system according to viability (Viable “green line” and non-viable (red line). A) Morphokinetic parameters of embryonic divisions t2, t3, t4, t5, t8, t9+, tM, tSB, tB and tBX; B) Time of cell cycle and synchrony t3-t2, t4-t3, t5-t4, t8-t5, t9+-t8, tM-t9+, tSB-ttM, tB-tSB, tBX-tB. (*) It indicates that in this morphokinetic parameter there is a statistical difference (P<0.05); Scatter plot illustrating two variables from the morphokinetics data, where colour represents *in vitro* viability, C) t2 (h) division time to 2 cells; D) Embryo diameter at tSB; and E) Embryo diameter at day 7.5 post IVF, with tSB (h) time of starting blastulation, respectively.

In Fig 3A and 3B, the morphokinetic parameters of embryonic development were classified and compared statistically according to *in vitro* viability. The variables tM, tsB and tBx (Fig 3A) and tm-t9+ (Fig 3B) showed statistical differences (P<0.05). Scatter plot showed that embryos with time of starting blastulation (tSB) less than 155 h maximized their in vitro viability to 54.54% (Fig 3C, 3D and 3E), although embryos with time of starting blastulation (tSB) greater than 155 h reduce to 6.67% their viability (see S1 Table). In Fig 3E, we show that a combination between time of starting blastulation (tSB) less than 155 h and embryo diameter measured at day 7.5 post IVF greater than 180 μm ensures maximum viability of *in vitro* produced blastocysts.

**Fig 4:**
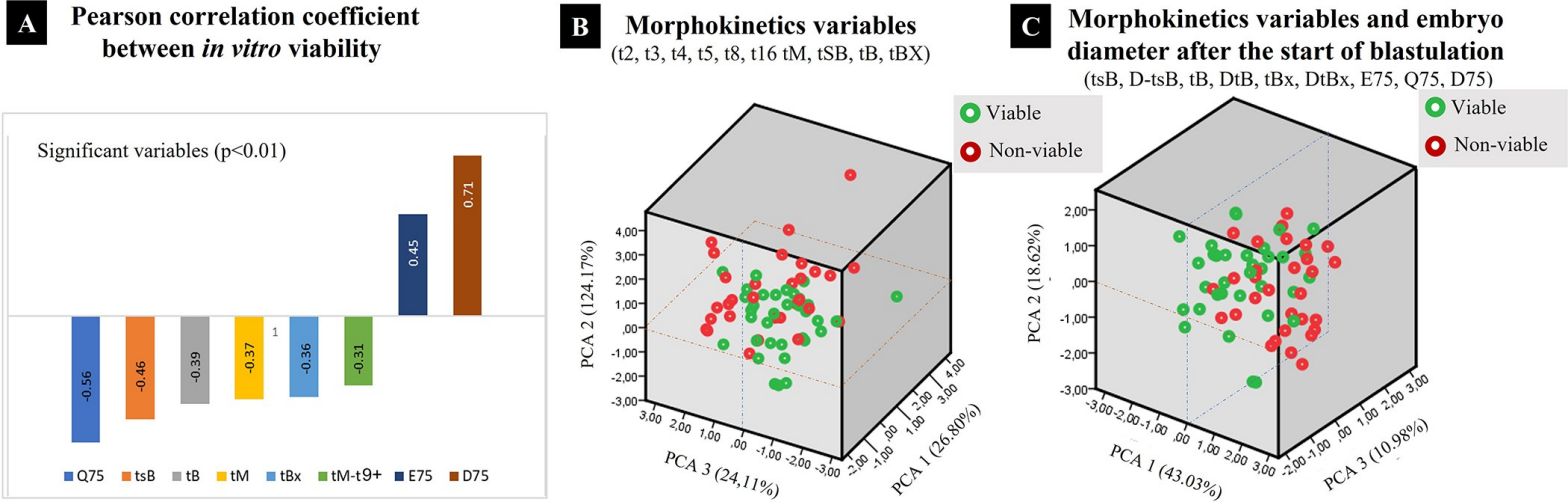
Pearson correlation coefficient and principal component analysis for all variables with *in vitro* viability. A) Variables con Pearson’s correlation coefficient significative (p<0.01). PCA analysis of morphokinetics variables (B) and morphokinetics variables and embryo diameter after the start of blastulation (C) according to their in vitro viability (V = viable and N = non-viable).

In Fig 4A, *in vitro* viability shows a positive and significant Pearson’s correlation coefficient with embryo diameter at day 7.5 post IVF (0.75) and a negative and significant correlation with embryo quality at day 7.5 post IVF (-0.56). Also, in Fig 4B and 4C, PCA analysis of multivariable data sets does not allow to emphasize variation or highlight patterns or cluster embryo morphokinetics variables (Fig 4B) and morphokinetics variables and embryo diameter after the start of blastulation (Fig 4C) according to their in vitro viability.

### Mathematical model to predict viability *in vitro*

Mathematical model-1 and model-2 included univariable data of the time of starting blastulation (tSB) and blastulation time (BT) had a fair predictor (ROC-AUC 0.7–0.8) of viability. While, the mathematical model-3 included the univariate data of embryo diameter at day 7.5 post IVF (D75) and had an excellent predictor of viability (ROC-AUC 0.92) (Tables 4 and 5). Additionally, the mathematical model-5 included four quantitative parameters of blastocyst (DtSB, DtB, DtBX, D75) after blastulation time and had the highest precision for predicting viability (ROC-AUC 0.93). Mathematical models 3, 4 and 5 showed a high ROC-AUC value >0.90 (Table 5).

**Table 4:** Non-invasive predictive models of *in vitro* viability using binary logistic regression.

Variable	Model-1 (tSB)	Model-2 (BT)	Model-3 (D75)	Model-4 (E75, Q75, D75)	Model-5 (DtSB, DtB, DtBX, D75)
Constant	15.274	2.830	-24.393	-24.11	36.014
**Blastocyst kinetics**					
BT (early = 1, late = 2)	--	-2.287	--	--	--
tSB	-0.106	--	--	--	--
**Blastocyst morphometry**					
D-tSB	--	--	--	--	-0.008
D-tB	--	--	--	--	-0.052
D-tBX	--	--	--	--	-0.364
E75	--	--	--	-0.038	--
Q75	--	--	--	-0.025	0.216
D75	--	--	0.125	0.125	--
**Algorithm power**					
ROC-AUC (0.9–1 = excellent, 0.8–0.9 = good, 0.7–0.8 = fair predictor)	0.79	0.75	0.92	0.92	0.93
Correct prediction (%)	70.8	74.0	81.3	81.3	82.8
Coef. Omnibus test	0.000	0.000	0.000	0.000	0.000
Nagelkerke R-Square	0.308	0.309	0.655	0.655	0.695
Cox and Snell R squared	0.227	0.228	0.484	0.484	0.521

Note: Time of starting blastulation (tSB), blastulation time (BT; early = 1, late = 2), time to blastocyst stage (tB), time to expanded blastocyst stage (tBX), diameter to time of starting blastulation (DtSB), diameter to time to blastocyst stage (DtB), diameter to time to expanded blastocyst stage (DtBX), embryo stage at day 7.5 post IVF (E75), embryo quality at day 7.5 post IVF (Q75) and embryo diameter at day 7.5 post IVF (D75).

**Table 5:** Logistic regression parameters of model-3 to predict viability *in vitro*.

Variable	B	95% CI de OR	P value
Constant	-24.393		0.000
D75	0.125	1.077–1.192	0.000

Regression coefficient (B), odds ratio (OR), Confidence interval (95% CI of OR).

### The logistic function (logit) for Model-3

Y=ln[p/(1‐p)]=‐24.393+0.125*D75

### The logistic function (logit) for Model-2

Y=ln[p/(1‐p)]=2.83–2.287*BT

### The logistic function (logit) for Model-1


Y=ln[p/(1‐p)]=15.274‐0.106*tSB

The estimated probability is:

p=ExpY/(1+ExpY)

Where:

ln is the natural logarithm, log exp, where exp = 2.71828…

ln[p/(1-p)] is the log odds ratio, or "logit"

p/(1-p) is the "odds ratio"

p is the probability that the event Y occurs, p (Y = 1)

If. p > 0.5, Predicts, embryo viable

p < 0.5, Predicts, embryo non-viable

Example: Estimated value of probability to predict viability for model 3:

**Table:** 

D75 (μm))	Y	p	Predicted
173.729	-2.68	0.06	Non viable
179.884	-1.91	0.13	Non viable
163.567	-3.95	0.02	Non viable
166.116	-3.63	0.03	Non viable
196.371	0.15	0.54	viable
158.534	-4.58	0.01	Non viable
164.131	-3.88	0.02	Non viable
194.569	-0.07	0.48	Non viable
201.487	0.79	0.69	Viable
229.554	4.30	0.99	Viable
202.061	0.86	0.70	Viable
211.086	1.99	0.88	Viable
213.196	2.26	0.91	Viable
231.816	4.58	0.99	Viable
225.276	3.77	0.98	Viable
235.291	5.02	0.99	Viable

## Discussion

Time-lapse system of embryonic development is currently one of the most advanced techniques used in *in vitro* embryo production in humans [31], cattle [32] and ovine [33]. In humans and bovines, morphokinetics has generated a database of parameters which has included the development of machine learning technologies to provide further insights to interpret and understand the embryonic development data.

The first division is an indicator of the developmental potential of embryos produced *in vitro* [34] and first division time is related to the state of polyadenylation and transcription of genes that are important for early embryonic development [35]. In this work, the average first cleavage time in bovine embryos was 31.18 h (Min 19.39 h and max: 69.33 h). Likewise, only 7.4% (2/27) of embryos cleaved after 36 h IVF developed into a blastocyst, similar to those reported by Dinnyés et al. [36] that showed very few blastocysts (5%) developed from embryos cleaved after 36 h IVF. Also, in human [37] and bovine [25] studies, time lapse monitoring has revealed that first cleavage after fertilization can be indicative of implantation potential. The first cleavage rate reported in this study was 83.65% (174/208), being similar to those reported by Rizos et al. [38] 84.3%, and Sanches et al. [39] 78.8%.

A conventional culture system (microdrops) allows the development of 30% of the oocytes matured *in vitro* to blastocysts [40–42] similar to the result reported in our laboratory control. However, the use of microwell culture to monitor embryonic development showed blastocyst rates greater than 30% in cattle [43] and humans [44–46] when cultured in a time-lapse system versus the traditional microdrops system. The difference in the higher rate of blastocysts in the time-lapse system could be due to the lower exposure to changes in pH, temperature, osmotic pressure and lighting during the observation and evaluation of embryonic development [47]. In addition, culture in a time-lapse system allows the non-invasive observation of key developmental markers, such as the extrusion of polar bodies, the formation of pronuclei, division times, and the duration of cell cycles that may be indicative of a greater potential of blastocyst development, which could greatly influence embryo selection by offering new opportunities and approaches for embryologists [48].

It is important to understand that a competent oocyte must develop to the blastocyst stage, and then, when transferred to the uterus of a recipient, it must have good interaction with the maternal environment and has the ability to implant. That is why during the last decades, optimizing *in vitro* systems that impact embryonic quality and competence has been a priority issue for laboratories and embryologists [49]. Likewise, non-invasive techniques that include observation or morphological and morphokinetic parameters for the classification of embryonic development are more frequently used in IVF programs [50,51]. In this study, embryos with *in vitro* viability have statistically different characteristics from non-viable embryos, being the key factor the speed of blastulation onset (Tables 2 and 3).

Post-hatching development (*in vitro* viability) in extended culture emerged as a more accurate alternative method to assess the development capacity of the embryo, without the need to be transferred to recipients and maintaining *in vitro* conditions similar to all embryos [52,53]. This stage of development can be used not only to assess the quality of embryos produced by different technologies, but also as a model to study embryonic loss during the period of cell differentiation and embryo elongation [53]. On the other hand, determining the viability of blastocysts under *in vivo* conditions has been carried out by transferring embryos at day 7 and recovering them after day 14 or 16 of development from the cow uterus [54–57]. Although this invasive technique may be more accurate in determining viability, the use of surgical procedures in the recovery of elongated blastocysts is poorly repeatable.

Our results show that post-hatching viability of embryos with early blastulation (63.27%; 31/49) was higher (P<0.05) than in late blastulation (14.87%; 7/47) embryos. Blastulation is an essential event in preimplantation embryonic development during which many molecular and morphological changes occur [58]. In addition, between the compact morula and blastocyst stages, the first differentiation of the cell lineage occurs, forming the inner cell mass (ICM) and the trophectoderm [59,60]. Likewise, early division favors the abundance of transcripts in all stages and increases blastocyst production [61], while early blastulation has been related to a better synchrony of embryonic development [18,62].

On the other hand, the use of time-lapse technology to monitor early development, pronuclear formation and fusion, and time to first division is quite common in humans [63,64]. However, the determination of the impact of the blastulation moment is little studied [65,66]. In humans, Ho et al. [14] and Moustafa et al. [16] indicated that time of blastulation has a high potential for embryo selection over other embryo morphologic grading components. Likewise, Lee et al. [15]. and Moustafa et al, [16]. reported that embryos with chromosomal abnormalities (aneuploid and mosaic) showed delayed blastulation. Nevertheless, in cattle, there are few reports regarding the moment of blastulation and its impact on subsequent in vitro development.

In humans, researchers have analyzed the prediction of embryonic morphokinetic evaluation (based on time-lapse system results) on implantation, results of combined mathematical models showed less precision than those shown in this work with ROC-AUC of 0.7 [67], 0.602 [66], 0.70 [68] and 0.71 [69]. Although, the ROC-AUC value was reduced to 0.561, when it only included the blastocyst morphology parameters [66]. However, models that consider morphokinetic variables are very useful for predicting blastocyst formation (ROC-AUC of 0.849). On the other hand, Alpha Executive and ESHRE Special Interest Group of Embryology (2011) proposed a blastocyst morphological evaluation system based on the combination of developmental stage and quality criteria. Even though, predictive models based on morphological parameters have low precision (ROC-AUC of 0.55) to predict successful pregnancy [70].

In cattle, some work has been done to predict pregnancy or implantation capacity in relation to morphological parameters and the time of the first embryonic division [71–73] although, the results have been variable and contradictory. Holm et al. [71] affirm that the time-lapse system is a superior method to study embryonic kinetics in cattle and to select embryos with a high probability of being competent (63 to 80%). On the other hand, Mellisho et al. [26]. used a mathematical model, with ROC-AUC value of 0.724, combining morphological and morphokinetic variables of the embryo (blastulation time, stage of development, quality, and diameter of the blastocyst at day 7.5) to predict *in vitro* viability. In this study, it was shown that the moment of blastulation affects the development of blastocysts diameter and quality at day 7.5 and *in vitro* viability.

The use of time-lapse technology has allowed the acquisition of morphokinetic parameters for the selection of viable embryos. These parameters include the duration of the first division of 1 to 2 cells, the time between division of 2 to 3 cells, the time between division of 3 to 4 cells, the cycle patterns of uniform divisions with short intervals in stage of 3 and 5 cells, and the time of the abrupt first cell division into 3 or more cells [23,22]. In humans, some studies report improved clinical outcomes when predictive morphokinetic models are used to select embryos for transfer [74], although the results are still controversial with other reports [22,48].

## Conclusion

The results from this work support that embryo morphokinetic variables at early stages of development could be used simply and routinely to predict developmental viability of in vitro produced bovine embryos. However, the high cost of time lapse monitoring equipment could limit its use in cattle. We propose two variables that require only two observations at the end of in vitro culture without the need for complex devices. Therefore, selection of blastocysts based on a blastulation time of less than 155 h and a blastocyst diameter measured on day 7.5 after IVF greater than 180 μm maximizes their viability in vitro.

## Supporting information

S1 Fig The morphokinetics parameters in embryo culture using time lapse system. (TIF)

S1 Table Morphokinetic development of bovine embryos. (XLSX)
