# Correction: Blastulation time measured with time-lapse system can predict in vitro viability of bovine blastocysts

**DOI:** 10.1371/journal.pone.0306750

**Published:** 2024-07-03

**Authors:** Carmen Huayhua, Misael Rodríguez, Jhorjhi Vega, Mario Briones, Lleretny Rodriguez-Alvarez, Edwin Mellisho

After publication of this article [1], concerns were raised that Fig 2 in [1] was duplicated from Figure 3 in a previous article [2] by a different author group, and that [2] was not cited in [1].

During editorial follow up on this issue, the corresponding author stated that Fig 2 in [1] was included in error.

Owing to the concerns about duplication with previously published content [2], published in 2010 by Oxford University Press [2], which is not offered under a CC BY license, this article [1] was republished on June 24, 2024, to correct the Fig 2 error. The updated figure reports demonstrative images of individual cultures in 16 microwell plates to monitor embryonic development in cattle. These images are from the original experiments reported in [1] at day 7.5 post IVF (S5 File). Replicate images from the time of the original experiments and images from earlier and later repeat experiments are provided here in S1–S4 Files. Additional data underlying S1 Table and supporting the embryo measurements in [1] are provided here in S6–S15 Files.

An member of the Editorial Board reviewed the concerns and stated that the updated Fig 2 supports what is stated in the published article, *‘During in vitro culture*, *16 presumptive zygotes were individually (Fig 2) monitored using the Primo Vision TL equipment (Vitrolife*, *Sweden) that takes images every 15 minutes from day 1 to 7*.*5 post IVF*.*’* [1]. They also advised that the data provided with this notice are sufficient to support the article’s key results as required by the PLOS Data Availability policy.

The corresponding author also provided some clarifying information about the methodology used in [1]:

- Time lapse was not used from day 7.5 to 9.5 but the culture was carried out under the same conditions (medium, gases and humidity). On day 9.5 after IVF, photographs of the hatched embryos were taken, archived in JPG format and processed with ImageJ software (S15 File).
- The Primovision equipment used in [1] has software that does not allow a recording time of more than 4 days, so in the experiment monitoring was carried out in two parts in the time lapse system, covering the time among presumptive zygotes at 7.5 days.
- Identification of time frames to division as part of the time-lapse video processing was done manually by marking points on the video (S14 File).

## Supporting information

S1 File Images from earlier repeat experiments supporting Fig 2. (JPG)

S2 File Images from later repeat experiments supporting Fig 2. (JPG)

S3 File Images from earlier repeat experiments supporting Fig 2. (JPG)

S4 File Replicate images supporting Fig 2 from the time of the original experiments. (PNG)

S5 File Images supporting Fig 2 from the time of the original experiments. (PNG)

S6 File Underlying data supporting the summary in S1 Table in [1]. (XLSX)

S7 File Supporting data for S1 Table and the embryo measurements in [1]. The times to divisions 2, 4, 8, etc. were processed individually, and the kinematics of the embryo was recorded in two parts because the Primovision (R) software did not allow continuous video recording for the 7.5 days post IVF. S7–S13 Files correspond to three batches of work.
(WEBM)

S8 File Supporting data for S1 Table and the embryo measurements in [1]. The times to divisions 2, 4, 8, etc. were processed individually, and the kinematics of the embryo was recorded in two parts because the Primovision (R) software did not allow continuous video recording for the 7.5 days post IVF. S7–S13 Files correspond to three batches of work.
(WEBM)

S9 File Supporting data for S1 Table and the embryo measurements in [1]. The times to divisions 2, 4, 8, etc. were processed individually, and the kinematics of the embryo was recorded in two parts because the Primovision (R) software did not allow continuous video recording for the 7.5 days post IVF. S7–S13 Files correspond to three batches of work.
(WEBM)

S10 File Supporting data for S1 Table and the embryo measurements in [1]. The times to divisions 2, 4, 8, etc. were processed individually, and the kinematics of the embryo was recorded in two parts because the Primovision (R) software did not allow continuous video recording for the 7.5 days post IVF. S7–S13 Files correspond to three batches of work.
(WEBM)

S11 File Supporting data for S1 Table and the embryo measurements in [1]. The times to divisions 2, 4, 8, etc. were processed individually, and the kinematics of the embryo was recorded in two parts because the Primovision (R) software did not allow continuous video recording for the 7.5 days post IVF. S7–S13 Files correspond to three batches of work.
(WEBM)

S12 File Supporting data for S1 Table and the embryo measurements in [1]. The times to divisions 2, 4, 8, etc. were processed individually, and the kinematics of the embryo was recorded in two parts because the Primovision (R) software did not allow continuous video recording for the 7.5 days post IVF. S7–S13 Files correspond to three batches of work.
(WEBM)

S13 File Supporting data for S1 Table and the embryo measurements in [1]. The times to divisions 2, 4, 8, etc. were processed individually, and the kinematics of the embryo was recorded in two parts because the Primovision (R) software did not allow continuous video recording for the 7.5 days post IVF. S7–S13 Files correspond to three batches of work.
(WEBM)

S14 File An example of how morphokinetics parameters were assessed in [1]. (PNG)

S15 File An embryo image of 9.5 post IVF (individual embryo culture) used to assess post-hatching development (in vitro viability). (PNG)

## References

[1] Huayhua C, Rodríguez M, Vega J, Briones M, Rodriguez-Alvarez L, Mellisho E (2023) Blastulation time measured with time-lapse system can predict in vitro viability of bovine blastocysts. PLoS ONE 18(8): e0289751. doi: 10.1371/journal.pone.0289751 37561791 PMC10414680

[2] Sugimura Satoshi, Akai Tomonori, Somfai Tamás, Hirayama Muneyuki, Aikawa Yoshio, Ohtake Masaki, Hattori Hideshi, Kobayashi Shuji, Hashiyada Yutaka, Konishi Kazuyuki, Imai Kei, Time-Lapse Cinematography-Compatible Polystyrene-Based Microwell Culture System: A Novel Tool for Tracking the Development of Individual Bovine Embryos, *Biology of Reproduction*, Volume 83, Issue 6, 1 December 2010, Pages 970–978, doi: 10.1095/biolreprod.110.085522 20739661

