# Supplementary figures and images for: Blastulation time measured with time-lapse system can predict in vitro viability of bovine blastocysts
Source: PLoS One. 2023 Aug 10;18(8):e0289751. doi: 10.1371/journal.pone.0289751 (PMC10414680; doi:10.1371/journal.pone.0289751)

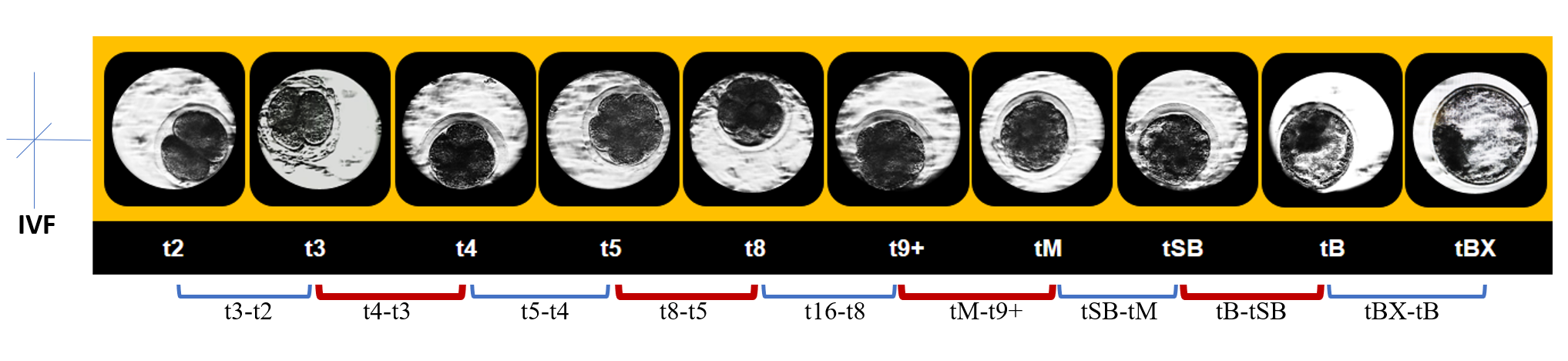

Supplement: S1 Fig — (TIF) [file pone.0289751.s001.tif]
