# Supplementary figures and images for: Correction: Blastulation time measured with time-lapse system can predict in vitro viability of bovine blastocysts
Source: PLoS One. 2024 Jul 3;19(7):e0306750. doi: 10.1371/journal.pone.0306750 (PMC11221745; doi:10.1371/journal.pone.0306750)

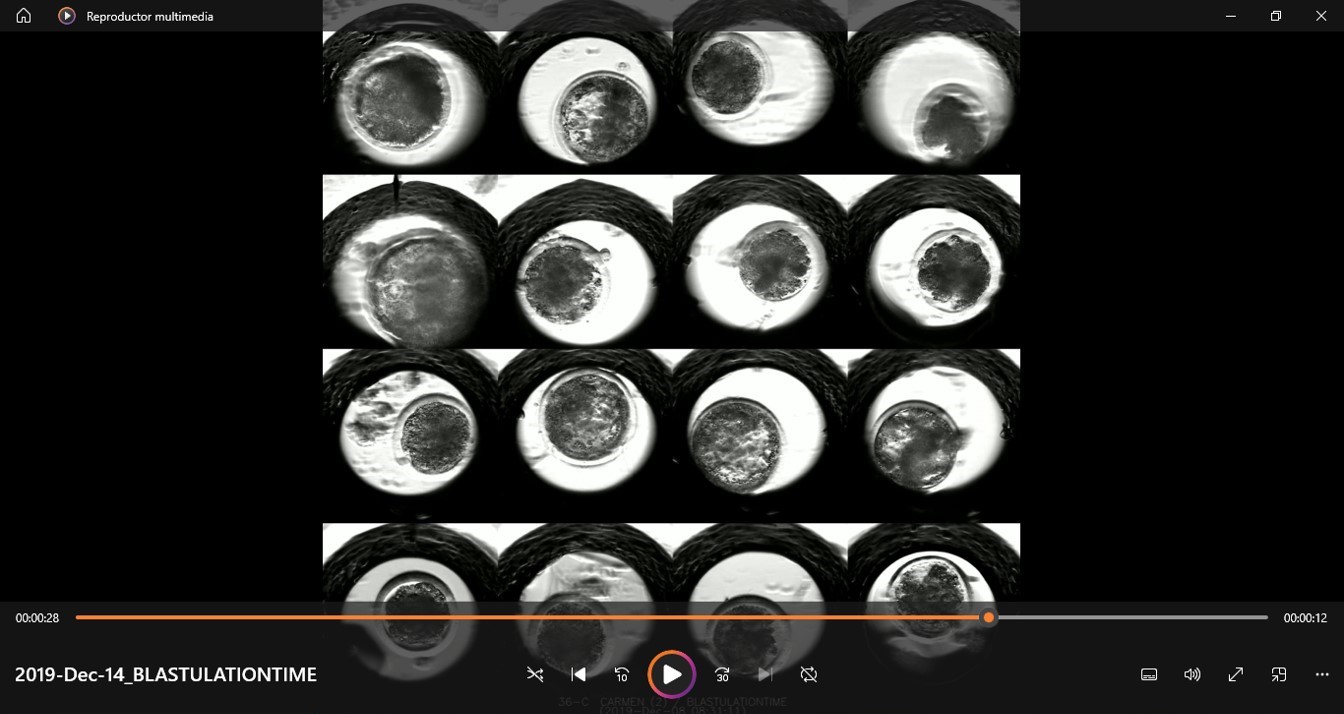

Supplement: S1 File — (JPG) [file pone.0306750.s001.jpg]

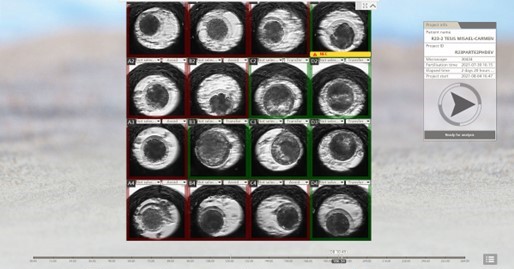

Supplement: S2 File — (JPG) [file pone.0306750.s002.jpg]

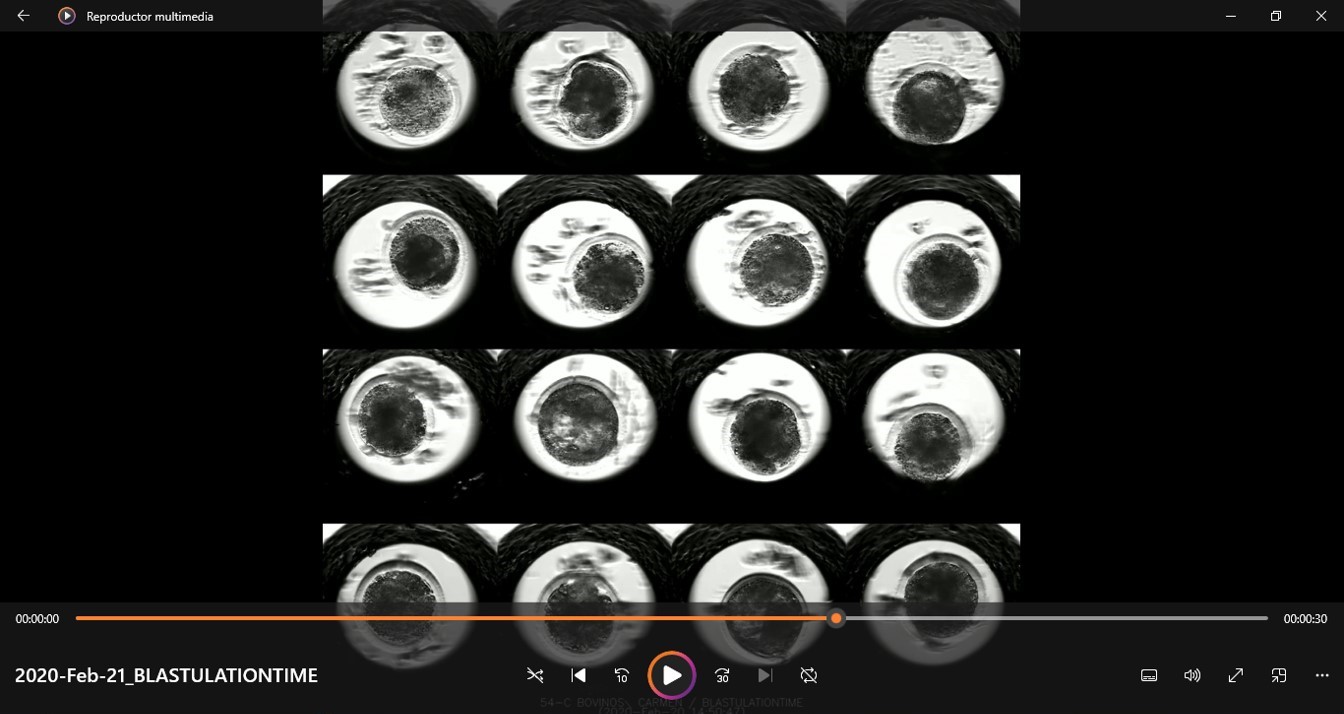

Supplement: S3 File — (JPG) [file pone.0306750.s003.jpg]

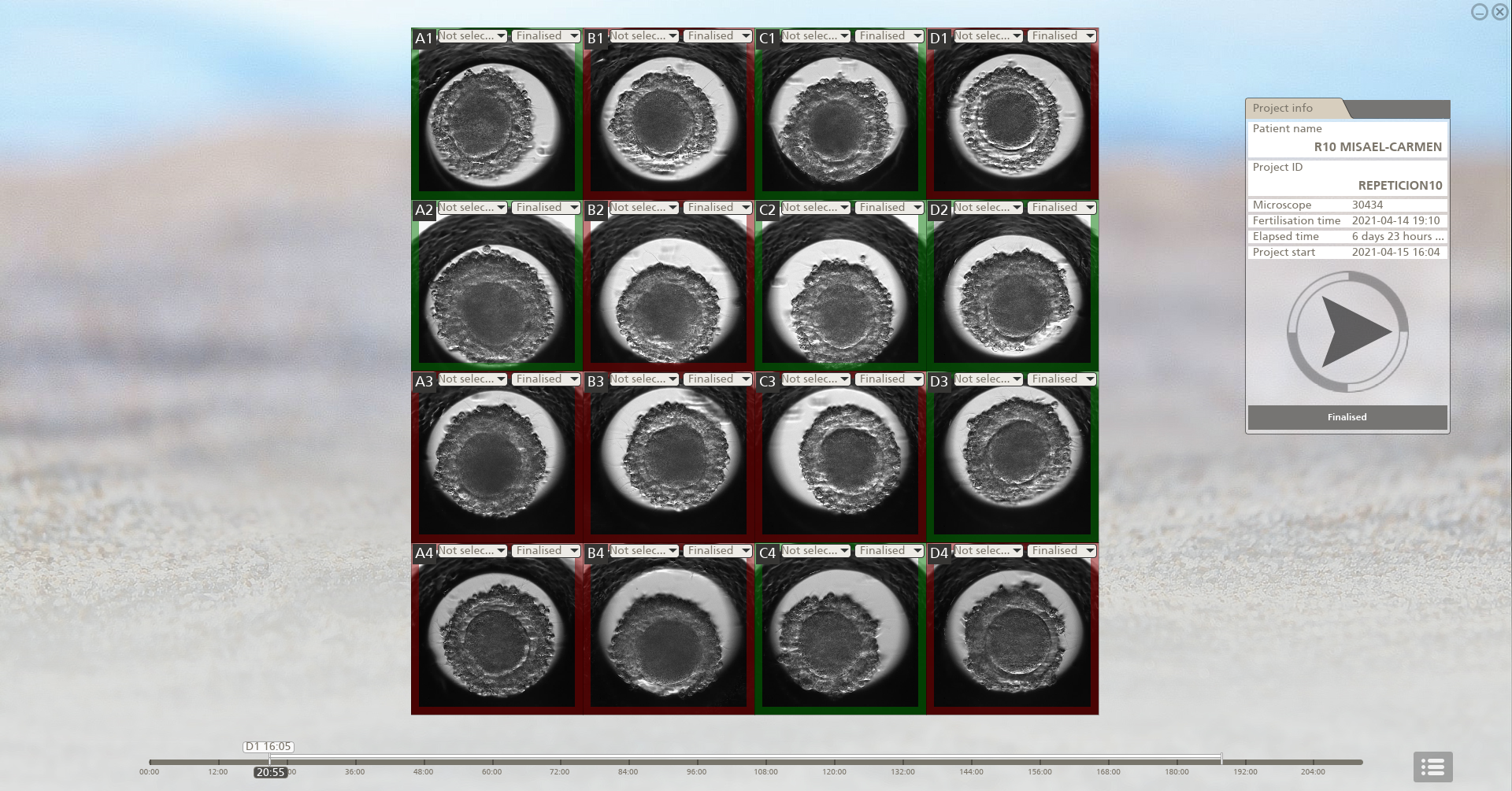

Supplement: S4 File — (PNG) [file pone.0306750.s004.png]

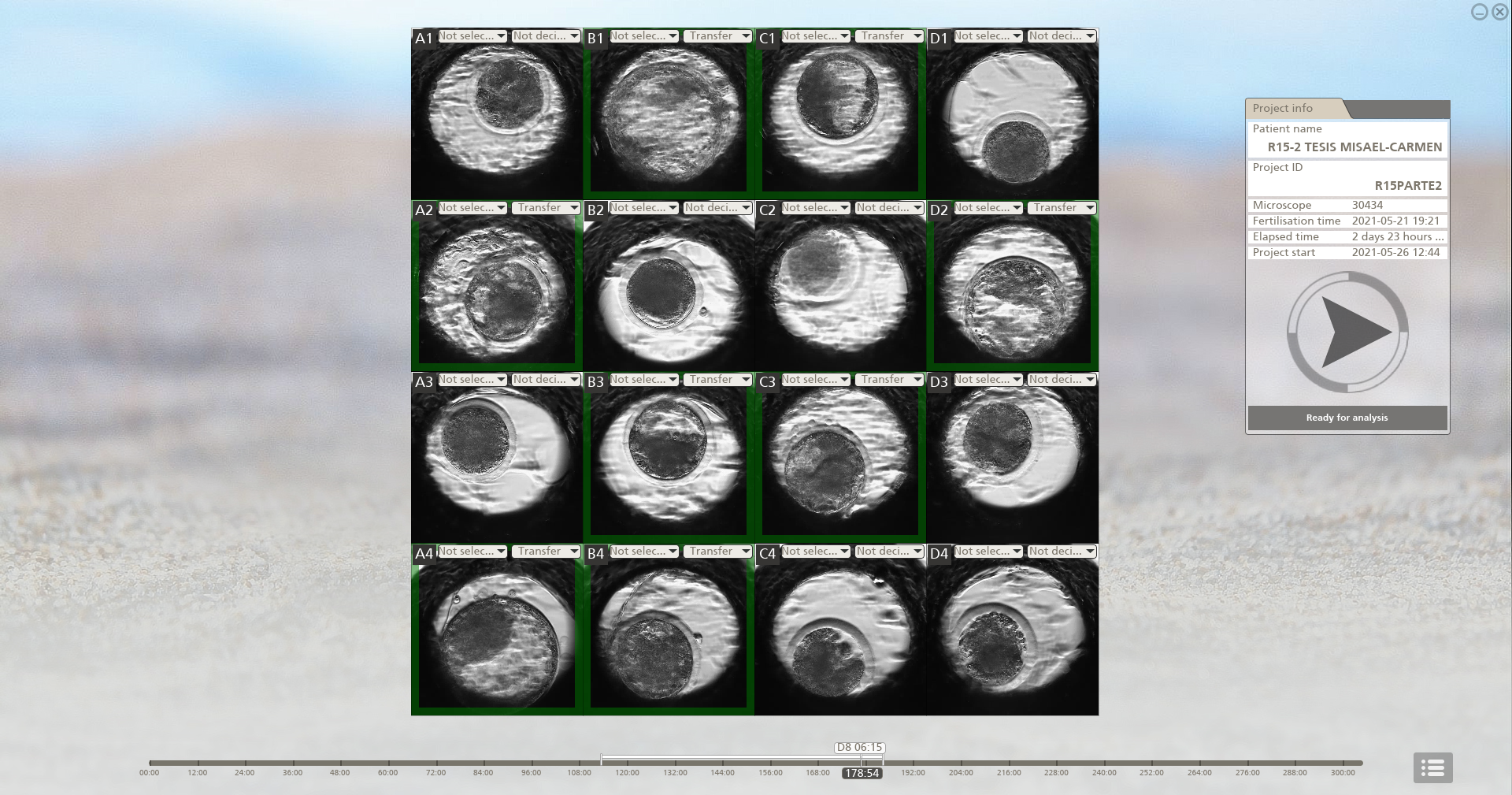

Supplement: S5 File — (PNG) [file pone.0306750.s005.png]

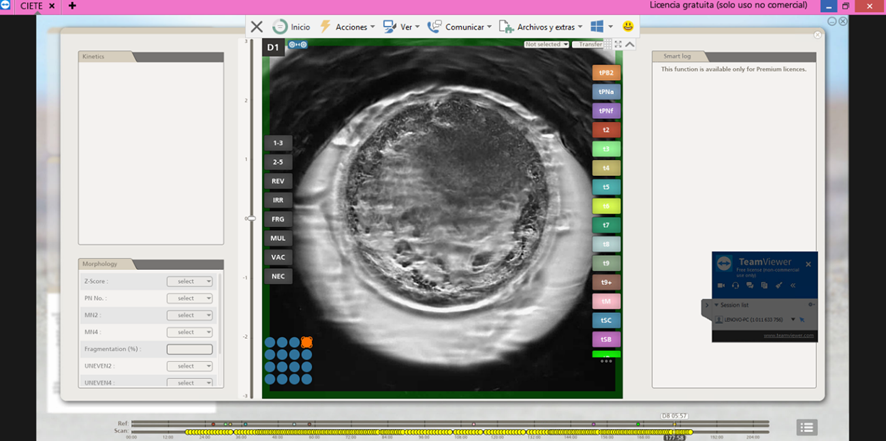

Supplement: S14 File — (PNG) [file pone.0306750.s014.png]

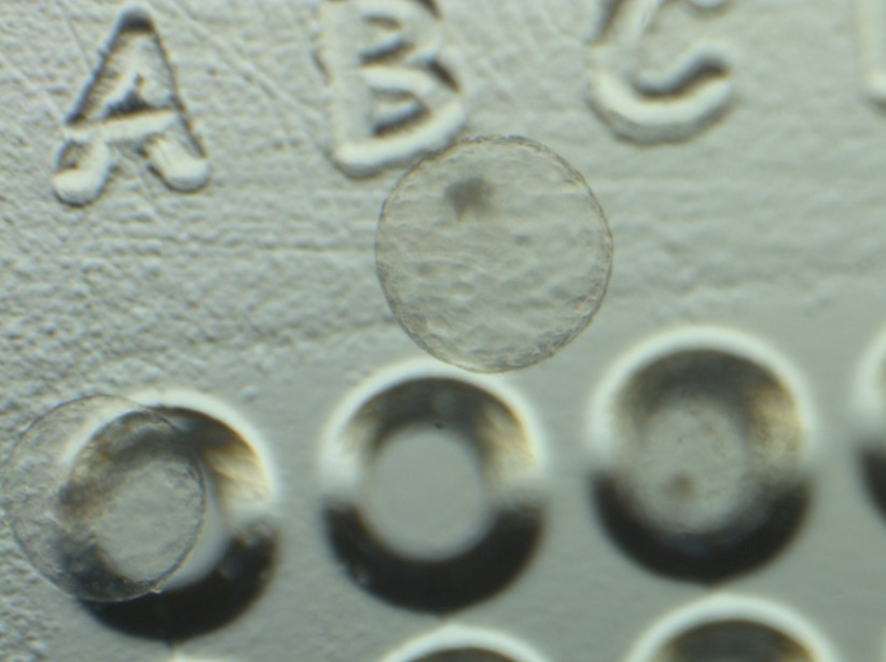

Supplement: S15 File — (PNG) [file pone.0306750.s015.png]
